# Inactivated H7 Influenza Virus Vaccines Protect Mice despite Inducing Only Low Levels of Neutralizing Antibodies

**DOI:** 10.1128/JVI.01202-17

**Published:** 2017-09-27

**Authors:** Ram P. Kamal, Kristy Blanchfield, Jessica A. Belser, Nedzad Music, Wen-Pin Tzeng, Crystal Holiday, Ashley Burroughs, Xiangjie Sun, Taronna R. Maines, Min Z. Levine, Ian A. York

**Affiliations:** a Battelle Memorial Institute, Atlanta, Georgia, USA; b Influenza Division, National Center for Immunization and Respiratory Diseases, Centers for Disease Control and Prevention, Atlanta, Georgia, USA; c Carter Consulting Inc., Atlanta, Georgia, USA; University of Kentucky, College of Medicine

**Keywords:** H7 avian influenza virus, immunogenicity, influenza, influenza pandemics, influenza vaccines

## Abstract

Avian influenza viruses of the H7 hemagglutinin (HA) subtype present a significant public health threat, as evidenced by the ongoing outbreak of human A(H7N9) infections in China. When evaluated by hemagglutination inhibition (HI) and microneutralization (MN) assays, H7 viruses and vaccines are found to induce lower level of neutralizing antibodies (nAb) than do their seasonal counterparts, making it difficult to develop and evaluate prepandemic vaccines. We have previously shown that purified recombinant H7 HA appear to be poorly immunogenic in that they induce low levels of HI and MN antibodies. In this study, we immunized mice with whole inactivated reverse genetics reassortant (RG) viruses expressing HA and neuraminidase (NA) from 3 different H7 viruses [A/Shanghai/2/2013(H7N9), A/Netherlands/219/2003(H7N7), and A/New York/107/2003(H7N2)] or with human A(H1N1)pdm09 (A/California/07/2009-like) or A(H3N2) (A/Perth16/2009) viruses. Mice produced equivalent titers of antibodies to all viruses as measured by enzyme-linked immunosorbent assay (ELISA). However, the antibody titers induced by H7 viruses were significantly lower when measured by HI and MN assays. Despite inducing very low levels of nAb, H7 vaccines conferred complete protection against homologous virus challenge in mice, and the serum antibodies directed against the HA head region were capable of mediating protection. The apparently low immunogenicity associated with H7 viruses and vaccines may be at least partly related to measuring antibody titers with the traditional HI and MN assays, which may not provide a true measure of protective immunity associated with H7 immunization. This study underscores the need for development of additional correlates of protection for prepandemic vaccines.

**IMPORTANCE** H7 avian influenza viruses present a serious risk to human health. Preparedness efforts include development of prepandemic vaccines. For seasonal influenza viruses, protection is correlated with antibody titers measured by hemagglutination inhibition (HI) and virus microneutralization (MN) assays. Since H7 vaccines typically induce low titers in HI and MN assays, they have been considered to be poorly immunogenic. We show that in mice H7 whole inactivated virus vaccines (WIVs) were as immunogenic as seasonal WIVs, as they induced similar levels of overall serum antibodies. However, a larger fraction of the antibodies induced by H7 WIV was nonneutralizing *in vitro*. Nevertheless, the H7 WIV completely protected mice against homologous viral challenge, and antibodies directed against the HA head were the major contributor toward immune protection. Vaccines against H7 avian influenza viruses may be more effective than HI and virus neutralization assays suggest, and such vaccines may need other methods for evaluation.

## INTRODUCTION

Avian influenza viruses of the H7 hemagglutinin (HA) subtype have sporadically infected humans on multiple occasions, usually causing mild disease (1, 2), and became a significant public health concern in 2003, when an outbreak of A(H7N7) highly pathogenic avian influenza (HPAI) viruses infected at least 89 people in The Netherlands, yielding one fatality due to severe pneumonia (3, 4). In addition to sporadic human infections (1), an outbreak of A(H7N9) avian influenza viruses in China, beginning in 2013, has infected over 1,552 humans, with a case fatality rate of nearly 40% (5).

Even in virologically confirmed human cases of H7 virus infection, titers of neutralizing serum antibodies (nAb) as measured by hemagglutination inhibition (HI) and microneutralization (MN) assays have often been undetectable or low compared to titers typically induced by human seasonal viruses (6–12). Similarly, efforts made to develop prepandemic vaccines against H7 viruses have been frustrating, as these have typically elicited poor HI and MN responses (13–18). Vaccines against human seasonal influenza A viruses (H1N1 and H3N2 subtypes) are most often composed of reassortant viruses, expressing the appropriate HA and neuraminidase (NA), that have been inactivated using compounds such as β-propiolactone (BPL). Such seasonal inactivated vaccines are capable of inducing HI titers of 40 or greater in recipients; a titer of 40 has been correlated with protection against seasonal influenza virus infection in 50% of the population cohorts (19–21). Protection against seasonal influenza viruses has also been suggested for an MN titer of 80 or more, as MN titers were found to be 2-fold higher than HI titers in general (22, 23). By extension from these well-studied viruses, it is believed that vaccines against H7 and other avian influenza viruses must also achieve HI titers of 40 or more to be effective. However, this assumption has not been tested with humans or animal models.

Especially since the A(H7N9) outbreak that began in 2013, efforts to develop prepandemic H7 vaccines have increased, including attempts to induce higher HI and MN responses using different manufacturing platforms (24–28), higher doses (15), and adjuvants (29, 30). Additionally, efforts have been made to explore components of H7 immunity other than neutralizing antibodies (31).

We have previously demonstrated that purified recombinant H7 HAs induced lower levels of neutralizing antibodies than did recombinant HA from seasonal viruses (32). However, it remained possible that recombinant HA does not accurately model whole inactivated influenza vaccines. Moreover, it was unclear whether the immune responses induced by recombinant H7 were actually protective, regardless of the HI and MN titers they induced. In the current study, we compared the antibody responses to three inactivated H7 viruses with those against two human seasonal influenza viruses and tested the ability of an inactivated A(H7N7) vaccine and passive transfer of immune sera to protect mice against infection with wild-type A(H7N7) virus.

## RESULTS

### H7 avian and seasonal human inactivated vaccines induce equivalent ELISA antibody titers.

**FIG 1:**
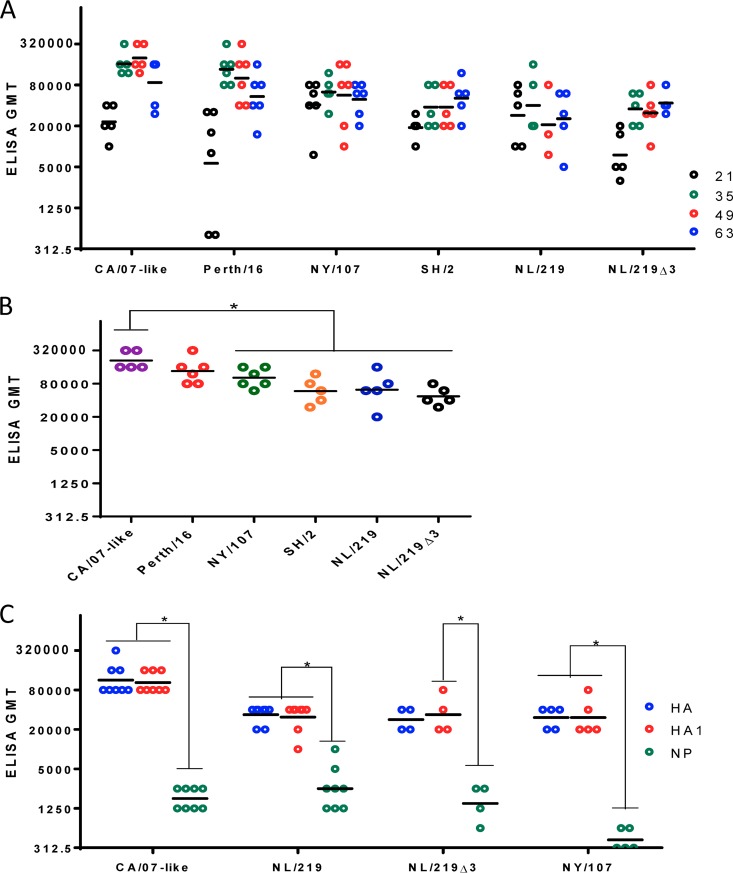
Mouse serum antibody titers as measured by ELISA. (A) Mice were immunized with inactivated purified viruses containing 3 μg of HA. On days 0, 21, 35, 49, and 63, serum anti-HA titers were measured by ELISAs, using purified recombinant homologous HAs as antigens. (B) Same experiment as in panel A, showing the maximum titers achieved by individual mice at any time point during the course of study. (C) ELISA antibody titers of day 35 sera from immunized mice used for challenge experiments. Recombinant homologous HA, HA1, and NP were used as coating antigens. Each symbol shows the titer of individual serum samples; the horizontal line indicates the group geometric mean. Statistical differences between groups are indicated by an asterisk (*P* < 0.05). Results from one representative experiment of 3 replicates are shown, except in the case of panel C (single experiment).

We used whole inactivated vaccine (WIV) preparations expressing HA and NA derived from three H7 viruses [(A/Shanghai/2/2013(H7N9) (SH/2), A/Netherlands/219/2003(H7N7) (NL/219), and A/New York/107/2003(H7N2) (NY/107)], one H1 virus [(A/Texas/5/2009(H1N1pdm09), antigenically identical to the prototypical H1N1pdm09 virus A/California/07/2009(H1N1pdm09) (CA/07) and henceforth referred as CA/07-like)], and one H3 virus [(A/Perth/16/2009(H3N2) (Perth/16)]. A mutant HA lacking three glycosylation sites on the head of NL/219 (33) (referred to as NL/219Δ3) was also used. To determine if H7, H1, and H3 viruses were comparably immunogenic in the mouse model, we immunized BALB/c mice twice at 3-week intervals and measured mouse IgG antibody responses to H7 and seasonal WIV immunogens by enzyme-linked immunosorbent assay (ELISA). Overall antibody responses of H7 WIVs by this assay were similar to those of seasonal WIVs (Fig. 1A). However, the kinetics of the ELISA-detectable antibody response differed between seasonal viruses, which achieved peak titers 35 or 49 days postimmunization, and the H7 WIVs, whose ELISA titers often continued to increase until day 63, although the differences in titers between the different days did not reach statistical significance (Fig. 1A). When only the maximum ELISA titers achieved by individual mice at any time point were considered, H7 WIVs all induced lower maximum ELISA titers than did CA/07 (*P* < 0.05) but were equivalent to Perth/16 (Fig. 1B). These data indicate that WIV preparations derived from the various influenza virus subtypes were capable of eliciting comparable anti-HA antibody responses in mice, as measured by ELISA.

ELISA titers against recombinant HA1 only (lacking the HA stem region) were equivalent to those against full-length recombinant HA (Fig. 1C), demonstrating that antibodies raised against H7 and H1 HA were predominately directed against the globular head of HA, rather than the more conserved stem region. Although the WIV used as the immunogen included viral proteins other than HA, the level of antinucleoprotein (anti-NP) antibodies in vaccinated mice was much lower than the level of anti-HA antibodies (Fig. 1C).

### H7 induces lower levels of HI antibodies than do seasonal viruses.

**FIG 2:**
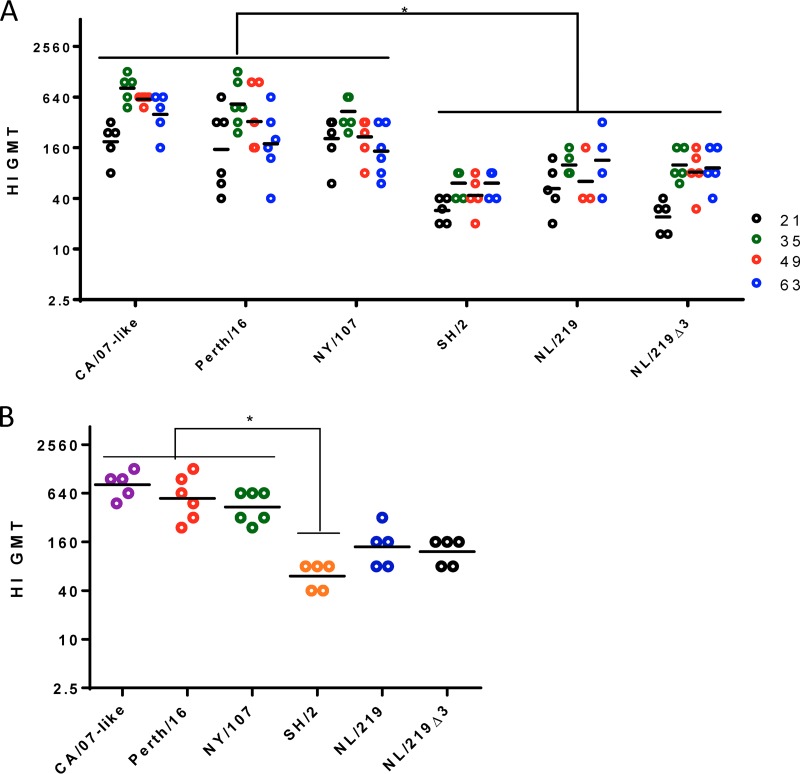
Mouse serum antibody titers as measured by HI. (A) Mice were immunized with inactivated purified viruses containing 3 μg of HA. On days 0, 21, 35, 49, and 63, serum anti-HA titers were measured by HI assays. (B) Same experiment as in panel A, showing the maximum titers achieved by individual mice at any time point during the course of study. Each symbol shows the titer of individual serum samples; the horizontal line indicates the group geometric mean. Statistical differences between groups are indicated by an asterisk (*P* < 0.05). Results from one representative experiment of 3 replicates are shown.

The HI assay is widely used to evaluate anti-influenza virus antibodies because it measures biologically relevant, protective antibodies that bind at or near the HA receptor binding site (RBS) and thus inhibit virus-induced hemagglutination. All mice immunized with CA/07-like and Perth/16 WIVs developed HI titers of 40 or more (a titer that for humans has been correlated with protection against infection with seasonal influenza viruses) after a single vaccination (Fig. 2), as did all six mice immunized with NY/107 WIV. However, only 7 of the 15 mice immunized with WIVs of NL/219, NL/219Δ3, or SH/2 achieved this titer after one immunization (Fig. 2A). After a boost with a second immunization, all mice, including those receiving H7 WIV, achieved HI titers of at least 40. Mice immunized with all H7 WIVs except NY/107 induced significantly lower HI titers than mice immunized with seasonal WIVs (Fig. 2A). A similar pattern was seen when considering only the maximum titers achieved by individual mice during the entire study course, although the difference was statistically significantly lower only for SH/2 (Fig. 2B).

### H7 induces very low titers of nAb.

**FIG 3:**
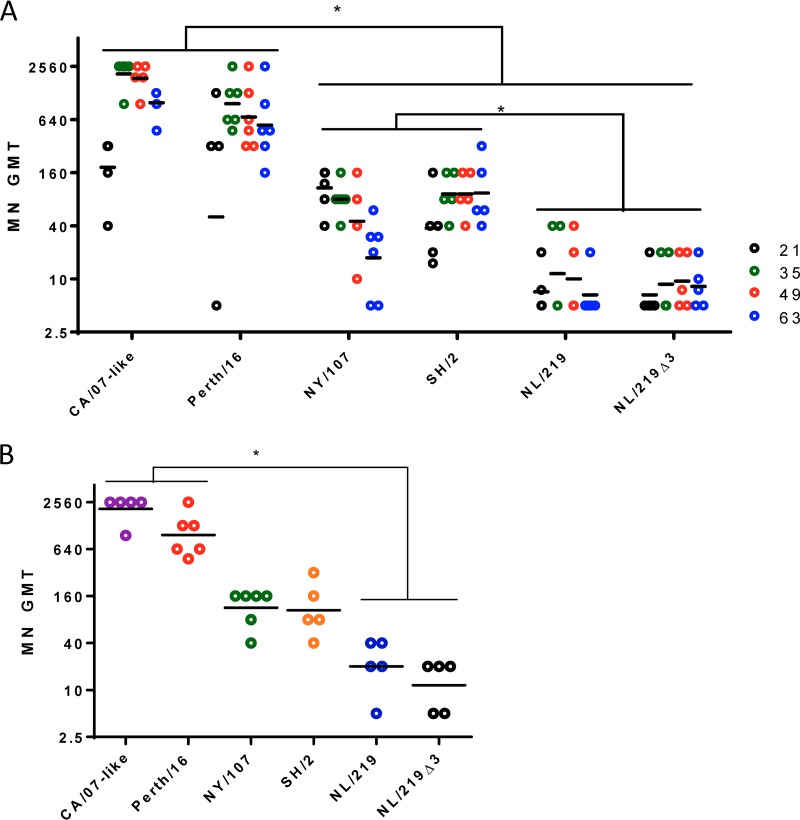
Mousee serum antibody titers as measured by MN. (A) Mice were immunized with inactivated purified viruses containing 3 μg of HA. On days 0, 21, 35, 49, and 63, serum anti-HA titers were measured by microneutralization assays. (B) Same experiment as in panel A, showing the maximum titers achieved by individual mice at any time point during the course of study. Each symbol shows the titer of individual serum samples; the horizontal line represents the group geometric mean. Statistical differences between groups are indicated by an asterisk (*P* < 0.05). Results from one representative experiment of 3 replicates are shown.

Microneutralization assays are designed to measure those antibodies which are capable of preventing virus infection in cultured cells (neutralizing antibodies [nAb]) and are therefore considered to measure the most biologically relevant antibodies. All mice immunized with seasonal WIVs developed high levels of nAb, achieving MN titers of 640 or higher 14 days after booster immunization. In 38 of 44 samples, the MN titer was equal to or greater than the corresponding HI titer. In comparison, 3 of the 10 mice receiving NL/219 and NL/219Δ3 WIVs did not develop detectable MN antibody titers, and none developed titers of 80 or more at any time point (Fig. 3A). While both NY/107 and SH/2 WIVs did induce detectable MN titers, in both cases these titers were significantly lower than those induced by CA/07-like and Perth/16 WIVs (*P* < 0.05) (Fig. 3A), in spite of the fact that NY/107 WIV induced titers in the HI assay and ELISA similar to those induced by the seasonal WIVs (Fig. 1 and 2). The pattern was similar when only maximum titers of individual mice were considered (Fig. 3B). Thus, all H7 WIVs induced significantly lower neutralizing antibodies titers than did seasonal (CA/07-like and Perth/16) WIVs.

### H7 induces higher proportions of nonneutralizing antibodies (non-nAb).

**FIG 4:**
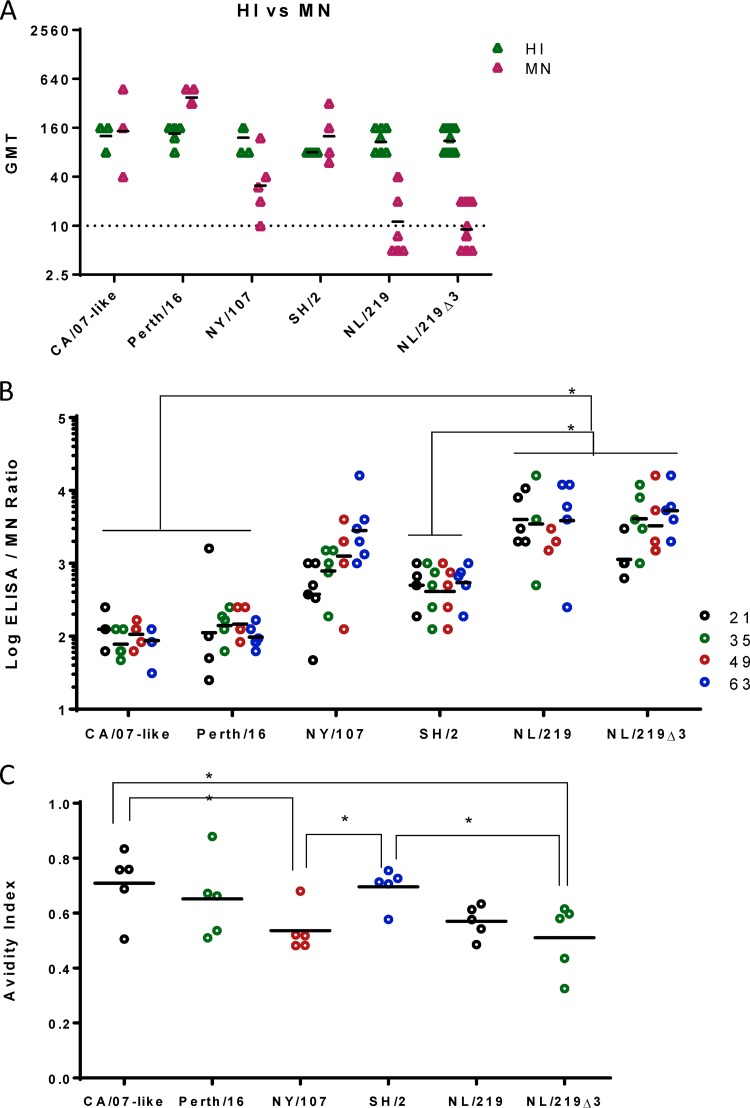
Estimation of non-neutralizing antibody titers. (A) MN and HI titers are shown from a subset of serum samples that had similar HI titers (80 to 160). (B) Nonneutralizing antibody titers were estimated by ratios of ELISA and MN titers. (C) Antibody avidity index of a subset of serum samples that had similar HI and ELISA titers. Each symbol shows the titer of individual serum samples; the horizontal line represents the group geometric mean. Statistical differences between groups are indicated by an asterisk (*P* < 0.05).

H7 and seasonal WIVs induced similar levels of overall IgG antibodies (as measured by ELISA), while H7 WIVs induced lower levels of antibodies that are traditionally considered to be most biologically relevant (as measured by HI and particularly MN). MN titers for seasonal WIVs are typically equal to or higher than their HI titers, as seen for Perth/16 and CA/07-like virus (compared Fig. 3A to Fig. 2A). Both HI and MN titers induced by H7 WIVs were generally lower than for seasonal WIVs. To more directly compare the MN to the HI titers induced by each WIV, we selected serum samples with similar HI titers (between 80 and 160) within each group and compared their MN titers. Of nine serum samples from mice immunized with CA/07-like and Perth/16 WIVs that had HI titers of 80 or 160, seven had MN titers that were higher than the HI titer (mean MN/HI ratio = 1.91). However, of the 34 serum samples from mice immunized with H7 WIVs that had HI titers of 80 or 160, 28 had lower MN than HI titers. The exceptions were samples from mice immunized with SH/2 WIV (Fig. 4A).

These observations suggested that a relatively large proportion of H7 antibodies might be nonneutralizing (non-nAb). We estimated the proportion of non-nAb by using a previously defined surrogate measure, i.e., the ratio of ELISA to MN titers (34). The highest proportions of non-nAb were found in recipients of NL/219 and NL/219Δ3 WIVs, while the CA/07 and Perth/16 WIVs induced the lowest proportions of non-nAb (Fig. 4B).

Antibody avidity could be one explanation for lower HI and MN titers detected in H7 antiserum. We measured antibody avidity of serum samples with similar HI titers by measuring the effect of a 4 M urea wash on ELISA optical density (OD) values (35, 36). The avidity of the NY/107 and the NL/219Δ3 antiserum was significantly lower than that of the CA/07-like antiserum. H7 antisera in general had lower antibody avidity than seasonal WIV groups, although the difference was not statistically significant for all groups. Again, the exception was SH/2 antisera, the avidity of which was equivalent to those for CA/07 and Perth/16 (Fig. 4C).

### H7 vaccines confer homologous protection.

The low levels of neutralizing antibodies induced by H7 WIVs raised the question of whether these responses would be protective against infection *in vivo*. Groups of 4 to 8 mice were immunized twice with CA/07-like, NL/219, NL/219Δ3, or NY/107 WIV as described above. Mice were challenged with wild-type homologous virus either 49 days (NL/219Δ3 and NY/107) or 63 days (CA/07-like and NL/219 groups) postimmunization (28 or 42 days postboost, respectively). NL/219Δ3- and NL/219-immunized mice were challenged with a lethal dose (50 and 100 50% lethal doses [LD_50_], respectively) of wild-type A/Netherlands/219/2003 virus (37). Those immunized with NY/107 WIV were challenged with 10^5^ 50% egg infective doses (EID_50_); since A/New York/107/2003 is not lethal in mice (37), it was not possible to use mortality as a measure of protection. Mice immunized with CA/07-like WIV were challenged with 10^5^ PFU of virus per mouse. The median MN titers of H7-immunized mice 2 weeks prior to challenge were 80, below the detection limit of 10, and below the detection limit of 20 for NY/107, NL/219, and NL/219Δ3, respectively. The median HI titers were 320, 80, and 80, respectively; and all the mice had ELISA titers of ≥10,000. The mice immunized with CA/07-like virus had higher median titers in the HI (640) and MN (2,560) assays.

**FIG 5:**
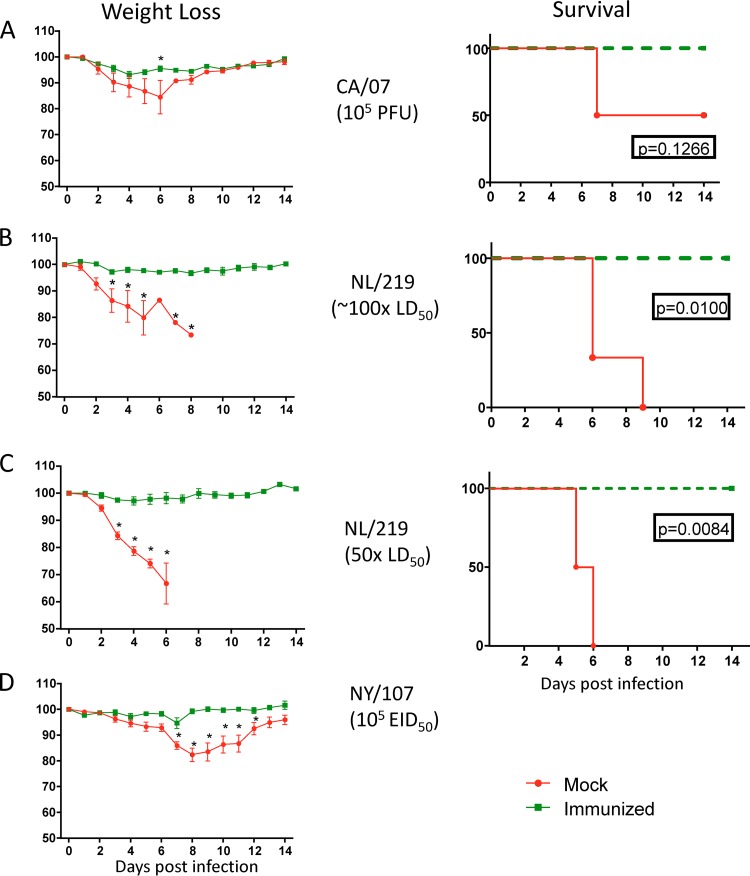
Morbidity and mortality in mice after homologous H7 challenge. Mice were immunized with CA/07-like (A), NL/219 (B), NL219Δ3 (C), or NY/107 (D) WIV. On day 49 or 63 postpriming (day 28 or 42 postboost), mice were challenged with homologous virus (infection doses shown). Mice were observed daily for morbidity and mortality for 14 days. The left graphs show the group mean weights ± SEM as a percentage of the day 0 weight. Daily statistical significance was calculated by multiple *t* tests. The right graphs show percent survival. Statistical significance between survival curves were calculated using the log rank (Mantel-Cox) test (*P* values are shown).

All unvaccinated control mice infected with wild-type A/Netherlands/219/2003 virus showed severe weight loss and were euthanized due to ≥25% weight loss by day 8 (NL/219) or day 6 (NL/219Δ3) (Fig. 5B and C). In contrast, none of the mice vaccinated with NL/219 and NL/219Δ3 WIV showed any noticeable sign of disease: all survived for 2 weeks postchallenge, with no significant weight loss (Fig. 5B and C). Similarly, mice vaccinated with NY/107 WIV showed no weight loss or other signs of disease, whereas unvaccinated control mice started losing weight on day 3 and lost about 15% of body weight by day 8, as well as showing other signs of illness (ruffled fur and hunched posture), but returned to normal body weight and showed no signs of illness by day 13 (Fig. 5D). Thus, both H7 WIV vaccines conferred complete protection in mice, in spite of the very low levels of nAb they induced.

**FIG 6:**
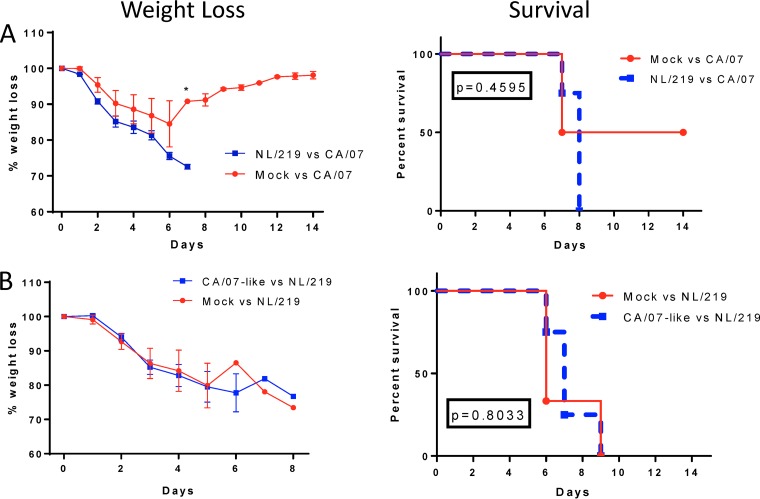
Morbidity and mortality in mice after heterologous challenge. Mice were immunized with NL/219 (A) and CA/07-like (B) whole inactivated vaccine. On day 63 postpriming (day 42 postboost), mice were cross-challenged with 10^5^ PFU of wild-type CA/07/2009 (A) or 8 × 10^2^ PFU of NL/219 (B) virus. Mice were observed daily for morbidity and mortality for 14 days. The left graphs show the group mean weights ± SEM as a percentage of the day 0 weight. Daily statistical significance was calculated by multiple *t* tests. The right graphs show percent survival. Statistical significance between survival curves were calculated using the log rank (Mantel-Cox) test (*P* values are shown).

In contrast to the strong homologous protection induced by the NL/219 and CA/07-like WIV, neither vaccine induced significant cross-protection. In cross-challenge experiments, mice immunized with NL/219 WIV and challenged with CA/07, or immunized with CA/07-like WIV and challenged with NL/219, all succumbed to the disease by day 8 or 9 (Fig. 6). This is consistent with the finding that the antibody response was predominately targeted against the globular head of HA, rather than the more conserved stem region or against conserved interval virion proteins such as NP.

### Serum antibodies against H7 HA confer immune protection.

**FIG 7:**
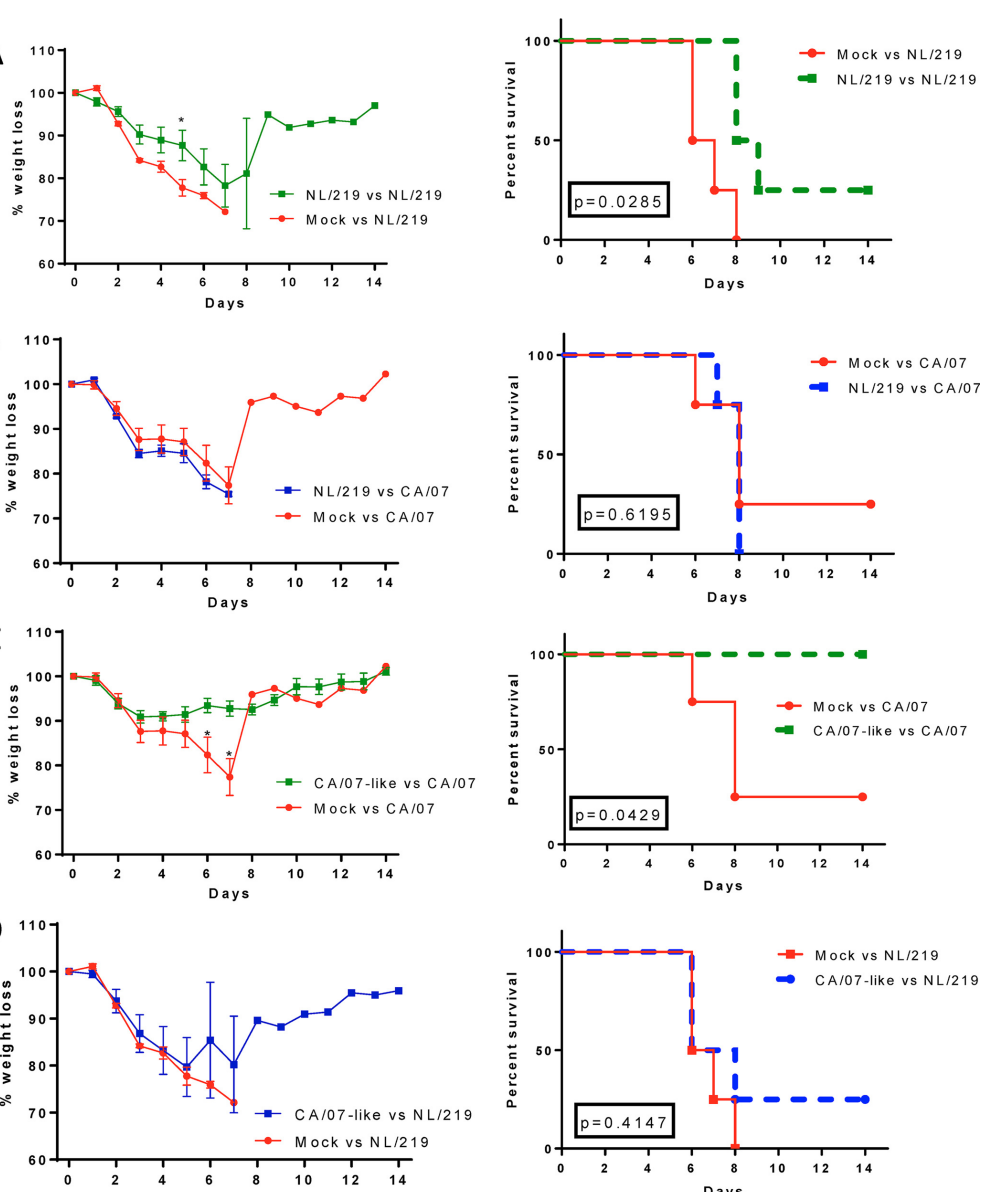
Passively transferred H7 and H1 sera confer homologous protection but no cross-protection. Two hundred microliters of pooled CA/07-like and NL/219 immune sera or nonimmune sera were passively transferred into naive mice. Twenty-four hours posttransfer, half of the mice in each group were challenged with 10^5^ PFU of A/California/07/2009 and the other half with 3.8 × 10^2^ PFU NL/219. Mice were observed daily for morbidity and mortality for 14 days. (A and C) Homologous protection; (B and D) heterologous protection. The left graphs show the group mean weights ± SEM as a percentage of the day 0 weight. Daily statistical significance was calculated by multiple *t* tests. The right graphs show percent survival. Statistical significance between survival curves were calculated using the log rank (Mantel-Cox) test (*P* values are shown).

To confirm that the protection observed after H7 WIV immunization was due to antibodies and not cell-mediated immunity, we transferred pooled serum from NL/219- and CA/07-like- WIV-vaccinated mice to naive mice and challenged the recipients with wild-type homologous and heterologous viruses. Recipients of serum from both WIVs were significantly protected against homologous challenge even in the absence of detectable nAb titers in NL/219 immune sera. As with active immunization, no heterologous protection was observed (Fig. 7). These experiments confirm that serum antibodies induced by H7 WIV, including nonneutralizing antibodies, primarily targeting the head of HA, are capable of mediating protection against homologous challenge and further rule out essential roles for the conserved internal virion proteins or for cell-mediated immunity in the protective responses observed in this study.

## DISCUSSION

Avian influenza viruses of the H7 HA subtype have proven their potential for future pandemics by two notable outbreaks: the 2003 H7N7 outbreak in The Netherlands and the ongoing H7N9 outbreak in China, now in its fifth wave. The continued detection of low-pathogenicity avian influenza (LPAI) and HPAI H7 viruses in North and South America, Europe, and Asia in recent years, necessitating the culling of millions of gallinaceous poultry, further highlights the potential risk of human exposure posed by H7 viruses worldwide. The public health approach to protection against influenza pandemics is through vaccination; there has therefore been significant interest in developing safe and effective vaccines against H7 influenza viruses.

The traditional approach to influenza vaccination is to use inactivated vaccines derived from reassortant viruses grown in embryonated eggs (38). This approach has proven reasonably effective against seasonal influenza viruses, against which inactivated vaccines are generally 50 to 60% effective (39). Protection against influenza is mainly associated with anti-HA antibodies, which can be measured by ELISA, HI, and MN. ELISAs quantify all binding antibodies, including those that bind to regions on the HA which are distant from the receptor binding site (RBS), and are capable of detecting lower-affinity antibodies than detected by HI or MN assays (40). However, ELISA titers required to achieve protection from infection are not defined (41, 42); in fact, high ELISA titers without high MN titers have been associated with increased severity of infection (34). HI assays measure antibodies that prevent interaction between HA and sialic acids on red blood cells and therefore measure those antibodies that bind near the RBS with high enough affinity to compete with sialic acids. MN assays detect functional antibodies that block virus infection and/or replication and thus are biologically relevant for protection against infection. For seasonal viruses, it has long been known that antibody titers of 40 or higher, as measured by HI assays, correlate with protection against infection (19–21). More recently, MN titers of 80 or more have been suggested to provide similar protection (24, 25).

Attempts to develop vaccines against H7 viruses have often not been successful in inducing HI titers that, in seasonal viruses, are correlated with protection (13–17). One reason for this may be that in humans, who have often been exposed to multiple strains of influenza virus through their lifetime, memory B cell responses may become focused on the conserved stem region; antibodies against this region do not mediate HI. However, we have previously shown that even in naive mice, recombinant H7 induces qualitatively different antibody responses than recombinant HA from human seasonal viruses (H1N1pdm09 and H3N2), with HA from H7 viruses inducing similar levels of antibodies (as measured by ELISA) but with lower levels of HI and MN activity, perhaps due to a lower-affinity response (32). However, it was not clear whether the immune response to purified recombinant protein would accurately reflect that to WIV. For example, the recombinant HA was expressed in insect cells, leading to different glycan profiles from the egg-produced WIV, and WIV contains low levels of viral components, such as genomic RNA, that may act as adjuvants (43). We therefore immunized mice with WIV derived from H7 (NL/219, NL/219Δ3, NY/107, and SH/2) and seasonal (CA/07-like and Perth/16) viruses and measured serum antibody titers using ELISA, HI, and MN.

As measured by ELISA, all six inactivated viruses induced similar titers of anti-HA IgG antibodies, suggesting that H7 is as capable of inducing specific antibodies as seasonal viruses. However, a large proportion of these antibodies were nonneutralizing, since MN titers were low and in many cases undetectable. HI titers induced by H7 WIV were also significantly lower than those induced by seasonal WIV. These observations are in accordance with a number of preclinical and clinical H7 vaccines studies that have also reported relatively low HI titers (6–9, 13–17). We therefore conclude that while H7 vaccines are technically as immunogenic as seasonal influenza vaccines, they induce a qualitatively different antibody response that has lower HI and MN components. Based on this and previous studies (13, 15, 32), this difference may be intrinsic to the H7 HA and is not overcome by the increased immunogenicity of WIV compared to that of purified recombinant HA. The molecular reasons for this difference are unclear. We had hypothesized that different levels of glycosylation might have been involved in the different response, as has been previously proposed for H3N2 influenza viruses (44–47); however, the antibody response to a mutant H7 containing three fewer N-linked glycosylation sites (NL/219Δ3) was very similar to that against the parental H7 (NL/219). Although it has been suggested that humans react less strongly to avian influenza virus HA than to seasonal virus HA because of their lack of priming to the former, in the present studies, mice with no previous exposure to any HA still produced lower MN and HI titers to the H7 WIV than to seasonal virus.

Although HI titer is well understood to correlate with protection for seasonal influenza viruses, it is not clear whether this assay also correlates with protection against avian influenza viruses such as H7. Indeed, in some cases, H7 vaccines have previously been shown to confer protection with low or no HI and MN titers (48, 49). Accordingly, we tested whether H7 WIVs were capable of protecting mice against wild-type viral challenge, despite the low HI and MN titers they induced. Unvaccinated mice challenged with NL/219 all developed severe disease and died by day 9 postinfection. However, mice vaccinated with NL/219 and NL/219Δ3 WIV were completely protected, without no sign of disease and no mortality. Unvaccinated mice challenged with wild-type A/New York/107/2003 virus showed significant weight loss, although as expected (37), none died. As with wild-type A/Netherlands/219/2003 challenge, all vaccinated mice were completely protected against weight loss. Even mice with no detectable MN antibodies (all NL/219 and NL/219Δ3 challenge group mice) showed complete protection against disease. Antibodies induced by the WIV were capable of mediating complete protection, since passive serum transfer from immunized to naive mice led to significant protection of the recipients. In addition, neither WIV conferred protection against heterologous influenza virus strains, as would be expected if T cells, antibodies against conserved internal proteins, or antibodies against the conserved stem region of HA played an important role in protection.

We therefore conclude that H7 WIVs may be capable of conferring protection against infection in spite of low HI and MN titers that are traditionally associated with protection in seasonal viruses. Our data, as well as other recent studies (31, 50, 51), suggest a significant role of non-nAb in protective immunity *in vivo*. It is possible that antibodies which *in vitro* are nonneutralizing may be protective *in vivo* against H7 infection, perhaps through interactions with complement (52–54) or accessory cells of the immune system (51, 55). It is intriguing to speculate that human-adapted viruses like A(H1N1)pdm09 or A(H3N2) have evolved some means of protection against some of these forms of inactivation, while avian-adapted viruses remain susceptible in their nonnative hosts. These findings suggest that additional correlates of protection should be explored in order to fully evaluate vaccines against avian influenza viruses instead of underestimating their potency based on lower HI and MN titers.

## MATERIALS AND METHODS

### Viruses and vaccines.

A/Puerto Rico/8/1934 (PR8) reverse genetics (RG) reassortants (2:6) of A/Shanghai/2/2013(H7N9) (SH/2; IDCDC-RG32A) (56) and A/Texas/5/2009(H1N1pdm09) (IDCDC-RG14) (57) have been previously described. Although the A(H1N1)pdm09 viruses originated as pandemic viruses in 2009, this strain has now become a circulating human seasonal virus. IDCDC-RG14 is antigenically identical to the prototypical H1N1pdm09 virus A/California/07/2009(H1N1pdm09) (CA/07) and is therefore referred as CA/07-like. A 2:6 PR8 reassortant containing the HA (lacking the polybasic cleavage site) and NA from A/Netherlands/219/2003(H7N7) (NL/219) was constructed using reverse genetics as described previously (58). A mutant HA lacking three glycosylation sites on the head of NL/219 (33) was constructed using standard molecular biology techniques to mutate Asn123 and Asn231 in HA1 and Asn82 in HA2 and was used with the wild-type NA to construct a PR8 reassortant (NL/219Δ3). An A/New York/107/2003(H7N2)-PR8 reassortant (NY/107) was kindly provided by Li-Mei Chen (CDC, Influenza Division). A 2:6 PR8 reassortant of A/Perth/16/2009(H3N2) (Perth/16; NIB64) was obtained from National Institute for Biological Standards and Control (NIBSC; London, UK). All PR8 reassortant viruses were propagated in 10- to 11-day-old embryonated chicken eggs, and allantoic fluid was frozen as virus stocks. Sequencing of virus HA, NA, and nonstructural (NS) segments was performed on each virus to confirm the absence of unexpected mutations in the stocks used. Whole inactivated virus (WIV) immunogens were prepared by inactivating allantoic fluid with β-propiolactone (Sigma-Aldrich, St. Louis, MO) at a dilution of 1:1,000 for 24 h, followed by purification by sucrose gradient ultracentrifugation. WIVs were tested for inactivation, which was confirmed by the absence of HA activity after 2 passages in eggs. Amounts of total protein and HA in the vaccines were measured using the Bio-Rad protein assay (Bio-Rad, Hercules, CA) and polyacrylamide gel electrophoresis (59), respectively, as described previously.

### Immunization, passive serum transfer, and protection studies.

Female 6- to 8-week-old BALB/c mice (Jackson Laboratory, Bar Harbor, ME) were used in this study. Groups of five or six mice were immunized intramuscularly (i.m.), twice at an interval of 3 weeks, with WIV immunogens containing 3 μg of HA. Control mice were mock treated with phosphate-buffered saline (PBS). Blood samples were collected on days 0 (preimmunization), 21 (preboost), 35, 49, and 63.

For *in vivo* homologous protection experiments, groups of four or five mice were prime boosted as described above with WIV from CA/07-like, NL/219, NL/219Δ3, or NY/107 virus. At day 49 or 63 postpriming (day 28 or 42 postboost), mice were challenged intranasally in a 50-μl volume with 10^5^ PFU of wild-type CA/07, 8 × 10^2^ PFU (∼100 50% lethal doses [LD_50_]) of wild-type NL/219 (37), or 10^5^ EID_50_ of wild-type NY/107 (37) virus and observed daily for morbidity and mortality for 14 days. Any mouse that lost ≥25% of preinfection body weight was humanely euthanized. For each virus, a control group of mock-vaccinated mice was also challenged. For heterologous protection, four mice each immunized with CA/07-like virus and NL/219 were cross-challenged with the same doses. Two weeks prior to challenge, serum samples were collected for antibody assays.

For experiments involving passive serum transfer, 12 mice were prime boosted with CA/07-like and NL/219 WIVs or saline (mock) as described above. Serum was collected by a terminal bleed on day 35. On the day of adoptive transfer, pooled sera were thawed, heat inactivated at 56°C for 30 min, and cooled to room temperature. Two hundred microliters of pooled immune or mock serum was injected intraperitoneally in eight mice per group. Half of the mice in each group were challenged with 10^5^ PFU of wild-type CA/07 virus and half with 3.8 × 10^2^ PFU (∼50 LD_50_) of wild-type NL/219 virus as described above.

All animal immunizations and blood collection were conducted at animal biosafety level 2 (ABSL2), while protection studies were done at animal biosafety level 3, including enhancements (ABSL3E), as required by the U.S. Department of Agriculture and the Federal Select Agent Program (60) at the Centers for Disease Control and Prevention (CDC). The protocols were approved by the CDC IACUC and were performed in an Association for Assessment and Accreditation of Laboratory Animal Care International-accredited facility.

### Antibody assays.

Postinfection ferret antisera raised in-house against homologous viruses were used as positive controls in all antibody assays (ELISA, HI, and MN). All sera (mice and ferret) were stored at −80°C, thawed immediately before assays, and treated with receptor-destroying enzyme (RDE; Denka Seiken, Tokyo, Japan) as described previously (61), resulting in an initial serum dilution of 1:10.

### ELISA.

ELISA was performed as described previously (62). Briefly, ELISA plates were coated with 1 μg/ml of recombinant HA, HA1, or NP protein obtained from the Influenza Reagent Resource (IRR) (Perth/16 HA [FR-472], Perth/16 HA1 [FR-835], NL/219 HA [FR-71], NL/219 HA1 [FR-844], CA/07 HA [FR-559], CA/07 HA1 [FR-695], and NY/107 HA [FR-69]). Recombinant purified NY/107 HA1 and SH/2 HA (63) (GISAID accession no. EPI439502) were kindly provided by James Stevens (CDC, Influenza Division). For NP ELISA, plates were coated with 1 μg/ml of PR8 NP (number 11675-V08B) and mouse monoclonal anti-NP (positive control) (number 11675-MM03T) obtained from Sino Biological Inc., China. Horseradish peroxidase (HRP)-conjugated goat anti-mouse IgG (number 115-035-164; Jackson ImmunoResearch Laboratories Inc., West Grove, PA) was used as a secondary antibody. *O*-Phenylenediamine (OPD) solution was used as the substrate, and the reaction was stopped after 10 min by adding 50 μl of 3 N HCl. Absorbance was measured at 490 nm. Antibody titers are given as the reciprocal of the highest serum dilution which gave an OD_490_ value greater than 2 times the average of the background wells.

### Avidity assay.

Antibody avidity was tested using a modified ELISA as described previously (32). Briefly, serum samples were added in duplicate to the antigen-coated ELISA plates. After incubation, one set of samples was incubated with 4 M urea (Sigma-Aldrich, St. Louis, MO) for 5 min, while the other set was incubated with standard wash buffer. Both sample sets were washed twice with wash buffer, and the absorbance (OD) was read. The avidity index (AI) was calculated as the ratio of (2 times the sum of ODs minus the ODs at the highest and lowest dilutions) (36) in ELISAs with and without the urea wash step.

### HI assay.

HI assays for seasonal viruses were performed as described previously (61). For H7 viruses, 1.0% horse red blood cells were used with a 1-h incubation time, while for seasonal virus HI, 0.5% turkey red blood cells were used with a 30-min incubation time. The detection limit of this assay was a titer of 10; samples with titers less than 10 were assigned a value of 5 for calculating geometric mean titers (GMT).

### MN assay.

MN assays were performed as described previously (61). The minimum detection limit of this assay was a titer of 10 (except in the case of NL/219Δ3 and NY/107 challenge mice groups, for which the limit was 20); samples with titers less than 10 were assigned a value of 5 for calculating geometric mean titers (GMT).

### Statistical analyses.

Statistical analyses for serum HI, MN, and ELISA titers were performed using a linear mixed model with repeated measures, implemented in SAS, using a cutoff of a *P* value of ≤0.05 for significance. Compound symmetry was used for the covariance structure, and 95% confidence intervals were also based on compound symmetry covariance to pool the variability among subgroups. For experiments that did not include repeated measures, statistical analyses were performed using GraphPad Prism 6. One-way analysis of variance (ANOVA) with Tukey's multiple-comparison test (maximal ELISA), the Kruskal-Wallis test with Dunn's multiple-comparison test (maximal HI and maximal MN and HI of immunized challenge mice), two-way ANOVA with repeated measures (nonneutralizing antibody [non-nAb]), and the Friedman test with Dunn's multiple-comparison test (ELISA Ab titers of vaccinated challenge mice against HA, HA1, and NP) were used. Survival curves were analyzed using log rank (Mantel-Cox) test, and daily body weight loss was analyzed by multiple *t* tests using the Holm-Sidak method for statistical significance. Antibody avidity differences between group pairs were compared using two-tailed unpaired Student *t* tests.
